# Combined healthy lifestyle factors are more beneficial in reducing cardiovascular disease in younger adults: a meta-analysis of prospective cohort studies

**DOI:** 10.1038/s41598-020-75314-z

**Published:** 2020-10-23

**Authors:** Ming-Chieh Tsai, Chun-Chuan Lee, Sung-Chen Liu, Po-Jung Tseng, Kuo-Liong Chien

**Affiliations:** 1 Division of Endocrinology and Metabolism, Department of Internal Medicine, Mackay Memorial Hospital, Taipei, Taiwan; 2 Department of Medicine, Mackay Medical Collage, New Taipei City, Taiwan; 3 Institute of Epidemiology and Preventive Medicine, College of Public Health, National Taiwan University, Room 517, No. 17, Xu-Zhou Rd., Taipei, 10055 Taiwan, ROC; 4 Division of Cardiovascular Surgery, Department of Surgery, Hsin Chu Armed Force Hospital, Hsinchu, Taiwan; 5 Department of Internal Medicine, National Taiwan University Hospital, Taipei, Taiwan

**Keywords:** Cardiology, Health care, Risk factors

## Abstract

To determine the association between combined lifestyle factors, including healthy diet, moderate alcohol consumption, non-smoking, physical activity, and optimal weight, and cardiovascular disease (CVD) risk among younger and older adults. We conducted a literature search using PubMed, EMBASE, Cochrane Library, and EBSCO databases up to November 30, 2019 and performed dose–response analysis, subgroup analysis and meta-regression with odds ratios and 95% confidence intervals (CIs). Twenty cohort studies involving 1,090,261 participants with 46,288 cardiovascular events and mean follow-up duration of 12.33 years were included. Compared with the group with the lowest number of healthy lifestyle factors, the group with the highest number had lower CVD risk [pooled hazard ratio, 0.37 (95% CI 0.31–0.43)]. With age as an effect modifier, the lifetime risk of CVD was 0.31 (95% CI 0.24–0.41) at age 37.1–49.9 years, 0.36 (95% CI 0.30–0.45) at age 50.0–59.9 years and 0.49 (95% CI 0.38–0.63) at age 60.0–72.9 years. The hazard ratio of CVD significantly increased from 37.1 to 72.9 years of age [slope in multivariate meta-regression: 0.01 (95% CI < 0.001–0.03; *p* = 0.042)]. Younger adults have more cardiovascular benefits from combined healthy lifestyle factors.

## Introduction

Cardiovascular disease (CVD) contributes to 31% of global deaths, and more than 75% of CVD deaths occur in low- and middle-income countries, where resources for medical care and further examinations to prevent premature mortality are limited. The proportion of worldwide cardiovascular deaths caused by heart attacks and stroke events is reported to be 85% according to World Health Organization. Life's simple 7 defined by American Heart association in 2010 recommends useful measures for the prevention of heart disease and stroke by modifying lifestyle behaviors^1^. Various healthy lifestyle behaviors, including healthy diet such as the Mediterranean diet^2–4^, alternative healthy eating index^5–7^, the recommended food score^8,9^, and others; moderate alcohol consumption^10^, non-smoking^11^, physical activity^12^, and maintaining optimal weight^13^ were reported to decrease the risk of CVD.

The benefits of combined healthy lifestyle habits in reducing cardiovascular events have been investigated in previous observational studies^2–9,14–26^, and a meta-analysis^27^. However, it remains unclear which basic characteristics of the population, such as mean age, sex proportion, the prevalence of diabetes at baseline, follow-up duration and ethnic groups, may be effect modifiers for the causal relationship between healthy lifestyle and CVD risk reduction. Although a previous study has reported the inverse relationship between CVD risk and combined healthy lifestyle habits among different age groups^25^, evidence from a systemic analysis is lacking. Therefore, the present meta-analysis aimed to determine the associations between combined lifestyle habits, including healthy diet, moderate alcohol consumption, non-smoking, physical activity, and optimal weight, and the risk of CVD among different age groups. Furthermore, we sought to examine whether age is an effect modifier for this causal relationship. Our research question is as follows: Will younger adults show greater reduction in CVD risk owing to combined healthy lifestyle habits than older adults?

## Material and methods

### Search strategy

Only prospective observational studies with relative risk (RR) of the relationship between combined healthy lifestyle behaviors and the incidence of CVD published until November 31, 2019 were included in our meta-analysis. We conducted systematic literature searches in PubMed, EMBASE, Cochrane Library, and EBSCO using the following search terms: “healthy lifestyle,” “risky health behaviors,” “combined effect,” “joint impact,” “cardiovascular disease,” “cerebral vascular accident,” “myocardial infarction,” “coronary artery disease,” “heart failure,” and “cardiac death”. The exact terms are described in Supplemental Table 1.

We identified additional relevant studies by manually searching and reviewing the reference lists of the retrieved articles. We limited our search to full-length, English language articles.

### Study selection (Supplemental Table 2)

The following three phases of the study selection process were performed: elimination of duplicated studies, selection of studies with related titles and abstracts, and full-text reading.

The inclusion criteria were as follows: (1) had a prospective observational study design; (2) the exposure of interest was combined lifestyle habits, including normal body mass index, healthy diet, physical activity, non-smoking, non-harmful alcohol consumption, and sedentary time (at least 3 lifestyle items); (3) the outcome of interest was the incidence of cardiovascular disease, including coronary heart disease (CHD), ischemic stroke, heart failure, or myocardial infarction; (4) participants were aged ≥ 20 years and had no history of CVD or other specific chronic disease; (5) risk ratio (RR) and confidence intervals (CIs) were reported or sufficient data were available to calculate them; and (6) the studies were published in a scientific journal and in English.

The exclusion criteria were as follows: (1) clinical trial or review article; (2) evaluation of a single or fewer than three healthy lifestyle behaviors or combined with other non-lifestyle factors, including blood pressure, blood glucose levels, or hyperlipidemia; (3) the definition of cardiovascular disease was not CHD, stroke, heart failure, or myocardial infarction; and (4) participants had CVD history.

When more than one study analysis presented different results from the same cohort, those, including longer follow-up durations and larger sample sizes were selected.

### Data extraction

According to the Meta-analysis Of Observation Studies in Epidemiology (MOOSE) guidelines^28^, four independent authors (MC. T., CC.L,. SC.L., PJ.T) extracted the data. The following data were collected from each study: authors, year of publication, cohort name, country, target population, sample size, mean age and sex distribution of participants, the prevalence of diabetes, person-year of different combined healthy lifestyle habit categories, definition of lifestyle habits (healthy diet, smoking status, alcohol consumption, physical activity, normal body mass index [BMI]), follow-up duration, main outcome, RR and 95% CI for all categories of combined healthy lifestyle habits, and adjusted habits . The authors of the original studies were contacted to obtain further details, such as RR and CIs, if these were not mentioned in their manuscripts.

### Quality assessment

The quality of the original studies that were included in the meta-analysis was evaluated using the Risk of Bias in Non-randomized Studies of Exposures (ROBINS-E tool)^29^. ROBINS-E includes the following seven domains of bias: confounding, selection of participants into the study, classification of exposures, deviations from intended exposures, missing data, measurement of outcomes, and selection of the reported result.

### Data synthesis

In the original studies, the effect size was commonly conducted by hazard ratio (HR). Some studies utilized the RR, which was regarded as interchangeable with HR. With regard to the various criteria for healthy or unhealthy lifestyle behaviors, the definitions dichotomizing lifestyle habits as optimal and not ideal, were according to each study. Combined healthy lifestyle habits were characterized by five main behaviors including healthy diet, moderate alcohol consumption, non-smoking, physical activity, and optimal weight. We pooled the HRs comparing participants with the highest adherence to the combined healthy lifestyle habits, with patients with the lowest adherence to represent the risk estimate comparing the ideal *versus* poor healthy lifestyle.

### Statistical analysis

Adjusted estimates of HR or RR from the original studies were used with approximately the same measurements. In the main analysis, the pooled effect size with 95% CI of the incident CVD between the categories with highest and lowest health behaviors were calculated using random-effects models by inverse-variance-weighted methods^30^ and fixed-effect models as a sensitivity analysis.

A linear dose–response analysis with the one-stage method (one-stage dose–response meta-analysis for aggregated data) and a non-linear dose–response analysis with the two-stage method were conducted to generate the study slope lines^31,32^. In the dose–response analysis, we enrolled studies reporting only five lifestyle behaviors, including healthy diet, moderate alcohol consumption, non-smoking, physical activity, and optimal weight, and excluded studies presenting > 5^6,16,22,24^ or < 5 lifestyle behaviors^2,3,5,7,17,18,20,21,25^ based on the score distribution of the study population. Working restricted cubic splines were used to analyze potential non-linear dose–response relations of the aggregated exposures.

Analysis of population subgroups classified according to age, a non-modifiable risk factor, was conducted to estimate the heterogeneity and mean effect size of expected patient proportions for all studies. The participants were divided into the following three age groups: 37.1–49.9 years, 50.0–59.9 years, and 60.0–72.9 years. We examined the modified effect with a sensitivity test stratified by different cut-off ages (37.1–49.9 and 50.0–72.9 years). We also investigated the age effect when the outcomes were different, including CVD, coronary artery disease (CAD), stroke, and heart failure. Separate random-effects regression analyses^33^ were performed for age, sex proportion, prevalence of diabetes, follow-up duration, and ethnic groups (European, American, and Asian) to clarify the potential influence of each basic characteristic. Furthermore, we used multivariate meta-regression models for estimating the slopes of combined healthy lifestyle habits and CVD risk as functions both containing age and female sex proportion with and without a cross-product term. In studies which collected the data across multiple years, the middle of the data range was used for our analysis. In addition, to illustrate the trend of evidence regarding the effect of combined healthy lifestyle habits on CVD, a cumulative meta-analysis^34^ was performed.

Publication bias was assessed by visual inspection of funnel plots and Egger's regression test for funnel plot asymmetry using the standard error as a predictor in mixed-effects meta-regression. We also measured the publication bias using the trim-and-fill method. Estimations of total heterogeneity, residual heterogeneity, and variability contributing to heterogeneity was measured using Cochran's Q test, tau^2^, and I^2^ statistics, respectively. All statistical analyses were conducted using R software (version 3.1, USA).

## Results

### Data extraction and study characteristics

The process of the literature search is illustrated in Supplemental Figure 1. A total of 20,808 studies were screened initially; of these, 20,786 were excluded because of duplication or they did not meet the inclusion criteria when checking the titles, abstracts, or full-texts. Twenty studies fulfilled the inclusion criteria and were included in the meta-analysis^2–9,14–25,35^. The four cohorts, namely the Swedish Mammography Cohort^2,9,15^, Cohort of Swedish men^2,8^, FINRISK cohort^19,20^, and the Women’s Health Initiative Observational Study (WHI-OS)^5,23^, provided results with different outcomes. We only included one of all studies from the same cohorts in the overall quantitative synthesis of the meta-analysis with longer follow-up duration and larger sample sizes. We considered all articles to be compatible with our criteria, regardless of whether they referred to the same cohorts. The characteristics of all 20 eligible studies are presented in Supplemental Table 3. All 20 studies had a prospective cohort design and were published between 2009 and 2019. A total of 46,288 cardiovascular events were included in the analyzed studies.

The cohorts included a total of 1,090,261 participants, and the mean follow-up duration across all studies was 12.33 years (6.2–22.4) years. All studies investigated at least three combined healthy lifestyle habits, including non-current smoking, non-harmful alcohol consumption, normal weight, healthy diet, and physical activity.

### Description of combined healthy lifestyle habits

Besides major lifestyle habits included in the combined lifestyle score, other lifestyle habits, such as television watching, afternoon nap habits, socialization, working hours, keeping normal waist-to-hip ratio, sleeping hours, medical checkups, and dental care were also considered in some studies^4,6,19,24^. Binary outcomes were created for each lifestyle habit, with 1 point representing a low risk and 0 points indicating a high risk. The combined healthy lifestyle score was the sum of the lifestyle points.

Healthy lifestyle habits were based on self-reports or data obtained via an interview. BMI was calculated using data on body weight and height, and most of the studies considered BMI of < 25 kg/m^2^ as a healthy lifestyle indicator with 1 point and BMI of ≥ 25 kg/m^2^ as an unhealthy lifestyle indicator with 0 points. The healthy lifestyle habit for tobacco smoking was classified as not currently smoking. Low alcohol consumption included alcohol consumption of 0.1–15 g per week for women^4^ and 0.1–30 g per day for men^8^, whereas high alcohol consumption was defined as exceeding 15 g per week for women and 30 g per week for men. Physical activity was dichotomized as ≥ 0.5 metabolic equivalent of task/hour/week^36^ as healthy, with 1 point, and < 0.5 MET/hour/week as unhealthy with 0 points. Healthy diet was defined as Mediterranean^2–4,37^, alternate healthy eating index^5–7^, recommended food score^2,8,9^ rich in vegetables, fruit, fish, whole grains, less meat^15,19,22,24^ and concentration of plasma vitamin C^18^.

### Descriptions of CVD

We defined CVD as myocardial infarction, CHD, ischemic stroke, and heart failure. We only included studies that presented the incidence of CVD as primary or secondary outcome via self-report or review of medical records. The effective estimation of combined healthy lifestyle and cardiovascular risk could be adjusted by using different variables, such as age, sex, time period, family history of CVD, aspirin use, hormonal therapy, age at menopause, and hypertension, diabetes mellitus, or hypercholesterolemia at baseline.

### Quality assessment

We conducted study quality assessment with risk of bias scores ranged from 0 to 4 (low-to moderate biases) according to ROBINS-E tool (Supplemental Table 4). Of the 20 studies included, 18 studies (90%) had a less than 4 bias risk. The most common risk was studies lacking information on the deviations from intended exposures and the attainment of exposure. The second bias risk was the variation in the degree of confounder adjustments, ranging from three to nine variables, with a mean of 6.9 (standard deviation ± 1.6) except in one study without a specific description in the article^16^. Most of the adjusted estimates were performed under the model contained data for age, sex, social-economic status, family history and chronic diseases at baseline. Nine studies^3,14,15,17–21,23^ considered clinical measurement of blood pressure and the lipid biochemistry marker in the adjusted model. Methods for confounding variables used in each study are presented in the supplement tables (Supplemental Table 3). The results indicated satisfactory methodological quality of the included studies.

### Publication bias

We generated funnel plots (Supplemental Figure 2), contour-enhanced funnel plots (Supplemental Figure 3), trim-and-fill (Supplemental Figure 4), and Egger Test (Supplemental Figure 5) to assess publication bias. Visual inspection of the funnel plot showed mild asymmetry. This was further confirmed by a significant Egger's test (*p* = 0.009).

### Meta-analysis

#### Overall cardiovascular risk

**Figure 1:**
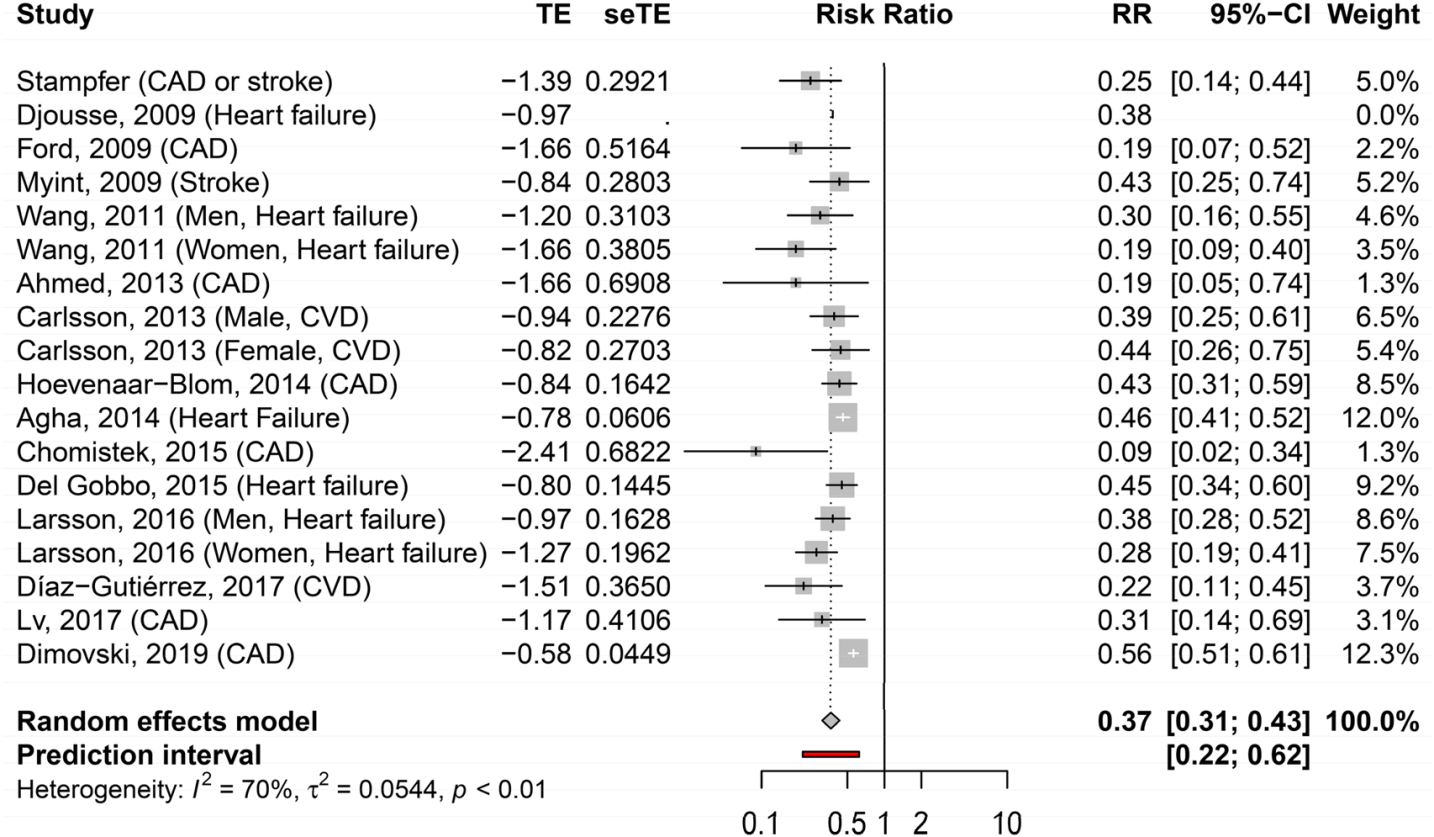
Forest plot of the adjusted hazard ratios with corresponding 95% CI of 15 studies on the association of combined healthy lifestyle and cardiovascular risk.

The meta-analysis including all studies reporting the incidence of CVD in those with the highest number of healthy lifestyle habits, compared to those with the lowest number, showed RR of 0.37 (95% CI 0.31–0.43) (Fig. 1). Between-study variation in terms of lifestyle definition and outcome measurement showed moderate statistical heterogeneity with an I^2^ of 70%.

#### Linear dose–response association

**Figure 2:**
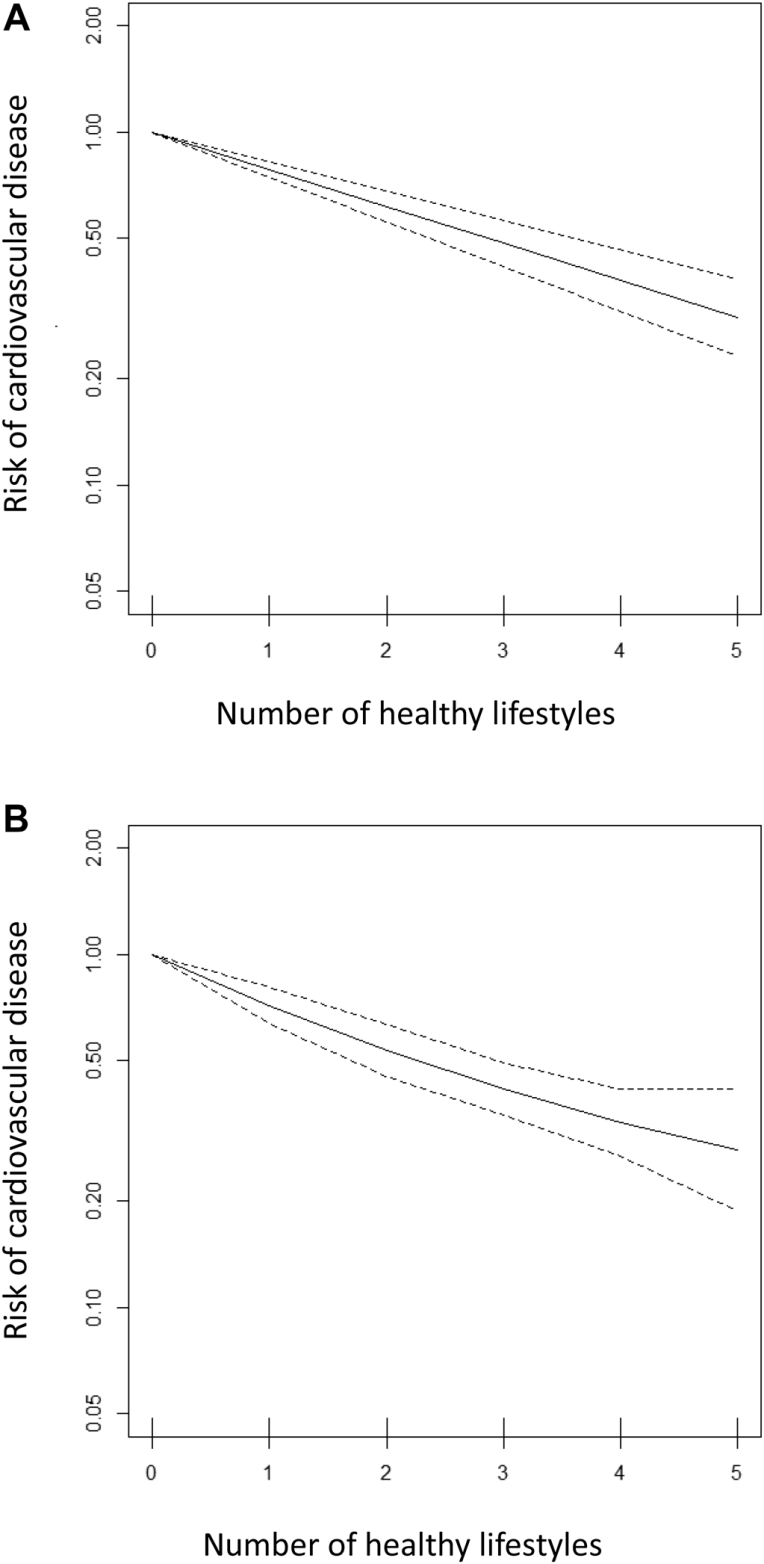
Dose–response relationship between the number of healthy lifestyle factors and incident cardiovascular disease; (**A**) Linear dose–response association (**B**) Non-linear dose–response association.

The pooled estimate from the linear dose–response meta-analysis was 0.79 (95% CI 0.75–0.83, *p* < 0.001) per 1-unit increase in the number of healthy lifestyle habits (Fig. 2A). Seven studies^6–8,14,19^ with 40 effect sizes were included in the non-linear dose–response analysis of the association between healthy lifestyle and cardiovascular risk. The non-linear association of the number of healthy lifestyle items with CVD was not significant (p value of non-linearity = 0.36) (Fig. 2B).

### Subgroup analysis

#### Subgroup on different definitions of outcomes

##### Subgroup analysis according to the different definitions of outcomes

A subgroup analysis was performed, and the pooled HRs were 0.27 (95% CI 0.19–0.39) (Supplemental Figure 6A), 0.39 (95% CI 0.33–0.46) (Supplemental Figure 6B), 0.37 (95% CI 0.30–0.46) (Supplemental Figure 6C), and 0.36 (95% CI 0.29–0.46) (Supplemental Figure 6D) for the outcome measurement of CHD, ischemic stroke, heart failure, and CVD, respectively.

##### Subgroup analysis according to age

**Figure 3:**
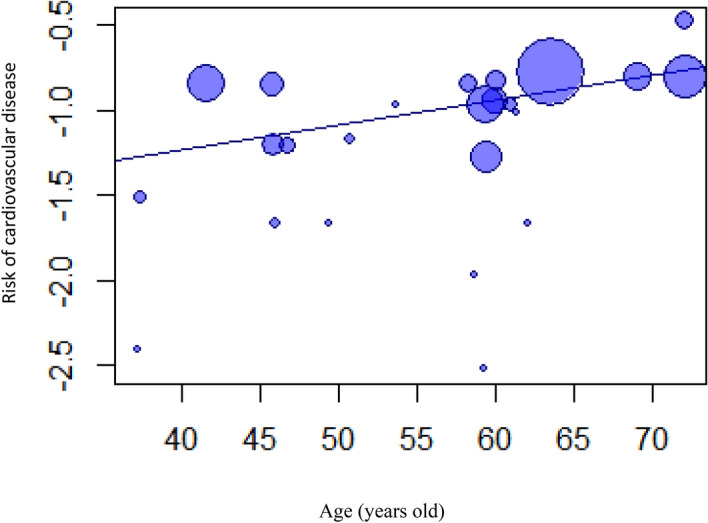
Age as a modifier factor on the preventive effect of cardiovascular disease demonstrated by meta-regression analysis. In these bubble plots, the size of a bubble is in proportion to the sample size of the corresponding study.

We found a significant correlation between combined healthy lifestyle habits and CVD risk among all age groups, with the 37.1–49.9-, 50.0–59.9-, and 60.0–72.9-year groups having an HR of 0.31 (95% CI 0.24–0.41), 0.36 (95% CI 0.30–0.45), and 0.49 (95% CI 0.38–0.63), respectively (Fig. 3). The trend of risk reduction was consistent among the subtypes of CVD (Supplementary Figure 7). The pooled HR (95% CI) was 0.14 (0.06–0.32) for the < 50-year subgroup and 0.19 (0.1–0.34) for the CAD subgroup. The pooled HR was 0.34 (0.22–0.53) for the < 50-year subgroup and 0.41 (0.34–0.50) for the stroke subgroup. The pooled was 0.25 (0.16–0.40) for the < 50-year subgroup and 0.41 (0.34–0.49) for the heart failure subgroup. The result from sensitivity tests by fixed-effect model were consistent with random-effect model.

##### Subgroup analysis according to the ethnic groups

The subgroup analysis according to the ethnic groups showed that all three subgroups had consistent cardiovascular protective effects from combined healthy lifestyle habits with HR (95% CI) of 0.46 (0.33–0.64) for American, 0.35 (0.30–0.41) for European, and 0.31 (0.14–0.69) for Asian ethnic groups (Supplemental Figure 8).

### Meta-regression of all characteristics

**Figure 4:**
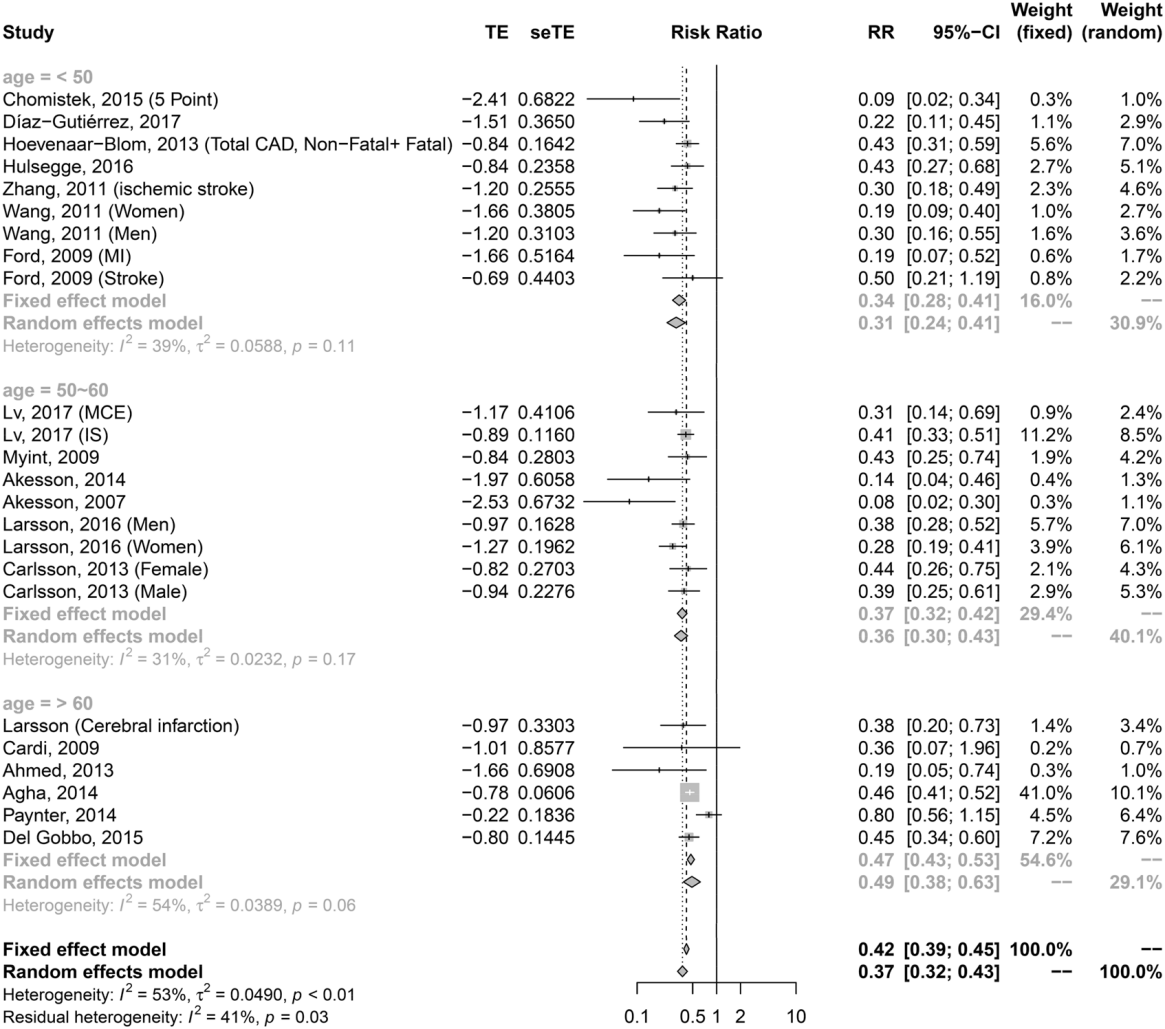
Forest plot of adjusted hazard ratios with corresponding 95% CIs of those with the maximal numbers of healthy lifestyle compared to those with the minimal numbers of healthy lifestyle and the incidence on different age groups: 37.1–49.9 years; 50.0–59.9 years; 60.0–72.9 years.

Owing to the significant between-study heterogeneity, several numbers of variables were tested to determine their influence on the relationship between combined healthy lifestyle habits and cardiovascular risk. The slope of combined healthy lifestyle habits and CVD risk gradually increased significantly in the univariate meta-regression models with age [slope: 0.01 (95% CI 0.002–0.03; *p* value = 0.028)] (Table 1; Fig. 3), which was consistent with the result of our subgroup analysis according to age that demonstrated a gradually increasing trend of HRs with increasing age (37.1–49.9, 50.0–59.9, and 60.0–72.9 years) (Fig. 4). More cardiovascular protective effects were observed in the population with a low prevalence of diabetes mellitus at baseline and longer follow-up duration, but both variables were insignificant in the univariate (Table 1) and multivariate meta-regression models adjusted for age and sex (Supplemental Table 5A,B and Supplemental Figure 8A,B). The ethnic and sex differences were not demonstrated in the association between combined healthy lifestyle habits and CVD risk reduction in the univariate meta-regression (Supplement Table 5C, D and Supplement Figure 9). Although women seemed to have more benefits from healthy lifestyle behaviors in terms of reducing the CVD risk compared to men when adjusting for age, the sex difference in the association between healthy lifestyle habits and CVD protection was insignificant in the multivariate meta-regression. However, age was consistently found to be an independent effect modifier of the association among different proportion of women (slope; 0.01 (95% CI  < 0.001–0.03; *p* value, 0.042)) (Supplemental Table 6A) that demonstrated young adults benefitted more than older adults from healthy lifestyle habits on cardiac protection among both sexes. It seems that the reduction of CVD risk from the combined healthy lifestyle habits among women was higher than that among men, but the difference was not significant (*p* value; 0.21). The age-related difference between women and men was also not observed (Supplemental Table 6B).

**Table 1:** Baseline characteristics as effect modifier factors between the association of combined healthy lifestyle factors and CVD reduction from the univariate and multivariate meta-regression model according to the age of study participants at baseline.

	Slope	95% CI		τ^2^ (%)	I^2^ (%)	*p* value
Univariate	0.01	0.002	0.03	1.2	18.73	0.028
Multivariate*	0.01	< 0.001	0.03	1.61	22.14	0.042

Multivariate model adjusted with sex.

τ^2^: the variance of the true effect sizes QM statistic and its *p* value show whether the moderator is statistically significant in explaining heterogeneity.

### Cumulative meta-analysis

As illustrated in Supplemental Figure 10, the trend of the association between combined healthy lifestyle habits and CVD risk has not significantly changed since 2013.

## Discussion

The present systematic literature review and meta-analysis demonstrated that combined healthy lifestyle habits reduce the development of CVDs. A major finding of this study is that the effect of healthy lifestyle behaviors can be observed in all age groups, but the preventive effect in adults aged 37.1–49.9 years was better than that of adults aged 60.0–72.9 years. The results indicate that the primordial prevention of healthy lifestyle showed more benefits in the younger population than in the older population. This finding may be due to the fact that healthy lifestyle behaviors as preliminary preventive measures for CVDs provide the best effects in younger and healthier individuals who have not experienced atherosclerotic changes yet. However, it remains unclear why healthy lifestyle has weaker effects on the elderly population.

Our findings are comparable with the results of the abovementioned empirical studies. With regard to the preventive effects of combined healthy lifestyle habits for CVD, our adjusted HR was comparable to that of a previous study reporting an adjusted HR of 0.34^27^, although there are important differences and robustness of findings if excluding 5 studies^8,9,15,19,23^ with the same cohort and including a latest study^25^ in 2019. These results lend some credence to the hypothesis that the effect of combined healthy lifestyle habits varies among different age populations^25^. The significant association found between healthy lifestyle and decreased CVD risk among different age groups seems to suggest that preliminary preventions are beneficial in all populations. This finding also reveals that the protective effects are consistent regardless of the sex distribution, prevalence of diabetes of the population, and follow-up duration. However, our data showed that the lowest HR from the combined healthy lifestyle habits on CVD was found in younger adults aged 37.1–49.9 years, whereas the highest HR was observed in the older adults aged 60.0–72.9 years. Moreover, the moderator analysis by both univariate and multivariate meta-regression demonstrated that age significantly impacted the cardiovascular protective effects of healthy lifestyle. Age as an independent modifier persisted in both women and men. Considering the study finding, the younger adults with a low short-term risk derived the most benefits from combined healthy lifestyles habits in terms of prevention of CVD compared to older adults with a high short-term risk.

The three possible reasons for age being an effect modifier may be the increased risk for atherosclerotic CVD with aging, low adherence to an ideal lifestyle among the elderly, and the legacy effect of non-optimal behaviors. A recent study suggested that 60% risk of 10-year predicted atherosclerotic CVD was attributed to aging alone^38^. The aging population has a higher HR of cardiovascular events than the younger population, regardless of lifestyle. Furthermore, modifiable lifestyle habits had less contribution to CVD risk, when including age as a significant factor^39^. The study data might explain why participants with advanced age experienced fewer benefits from modifiable lifestyle behaviors in terms of preventing cardiovascular events. Another reason for our finding was that poor lifestyle behaviors do not only alter the short-term risk of developing CVD, but also have a legacy effect^40^ on the long-term CVD incidence. The elderly adults were more likely to have engaged in non-ideal activities than younger individuals during their youth. Consequently, the elderly population also has a greater risk of CVD development, regardless of whether they previously engaged in healthy lifestyle behaviors in the recent years. Finally, individuals who have chronic diseases are more strongly motivated to maintain a healthy lifestyle. However, chronic disease might be a potential confounder in CVD development. Furthermore, a higher prevalence of chronic disease was noted in the elderly participants than in the younger participants. This may result in increased CVD risk among those with healthy lifestyle but with an underestimated chronic disease. Nevertheless, our study revealed greater benefits of a healthy lifestyle for younger populations than for the elderly.

There are several implications of our finding that age is an effect modifier of the association between healthy lifestyle scores and CVD risk, specifically before 60 years old. Identifying unhealthy lifestyle habits among young and middle-age adult with low short-term CVD risk and aggressively healthy lifestyle intervention is crucial for improving cardiovascular health at a population level. Additionally, different strategies separately for the primordial prevention from primary prevention, such as the reduction of clinical CV risk (i.e., hypertension, hypocholesteremia, and diabetes) were required. Regarding primordial prevention, combined healthy lifestyle habits have an important role to identify unhealthy habits proceeding the development of CVD. Finally, the absence of clinical measurements in combined healthy lifestyle habits is a more useful and economical strategy increasing the application in community-based or primary healthy service and even individuals at home without the available laboratory-based measures.

This meta-analysis has some strengths. We included a large number of studies with a large sample size and long follow-up period. We also performed a stratified analysis and provided novel estimates of the modifier effect of age in altering the reduction of CVD from healthy lifestyle. Moreover, important confounders were adjusted for in most of the included articles. The studies were of high quality as evaluated by using the ROBINS-E tool. Nevertheless, this study also has several limitations. Firstly, the high heterogeneity of the pooled estimates might result from the varying definitions of healthy lifestyle, outcome, outcome measurement, and the number of combined healthy lifestyle habits. Nevertheless, a consistent association between healthy lifestyle and cardiovascular risk was presented in the subgroup analysis. Further studies of specific lifestyle and outcome should be investigated. Secondly, publication bias was observed in our studies. The publication bias would be a consequence of the preference for submission and publication from authors, editors and reviewers and the influence of various factors on the decision-making^41^. We should persist in reporting all studies of a high quality, regardless of results, to overcome publication bias in the future.

## Conclusion

The present systematic literature review and meta-analysis found a decreased incidence of CVD in individuals with the highest number of combined healthy lifestyle habits (non-smoking, moderate alcohol consumption, healthy diet, physical activity, and optimal weight). Younger participants experienced more benefits from healthy lifestyle behaviors in terms of reduction of CVD risk than older participants. In clinical practice, more attention needs to be paid to the prevention, identification, and treatment of CVD in individuals with a low number of healthy lifestyle habits. Moreover, decreasing the CVD risk could potentially be achieved through promotion of modifiable health behaviors. Finally, our data could contribute to the establishment of personalized preventative measures and population-level interventions against CVD.

## Supplementary information

Supplementary Informations.

## Abbreviations

CVD: Cardiovascular disease
CHD: Coronary heart disease
BMI: Body mass index
HR: Hazard ratios
RR: Risk ratio
