# Supplementary material for: Combined healthy lifestyle factors are more beneficial in reducing cardiovascular disease in younger adults: a meta-analysis of prospective cohort studies
Source: Sci Rep. 2020 Oct 23;10:18165. doi: 10.1038/s41598-020-75314-z (PMC7584648; doi:10.1038/s41598-020-75314-z)

**Title:**

**Combined healthy lifestyle factors are more beneficial in reducing cardiovascular disease in younger adults: A meta-analysis of prospective cohort studies**

**Running title: Healthy lifestyle**

**Ming-Chieh Tsai^1,2,3^,** ,**Chun-Chuan Lee^1,2^ ,Sung-Chen Liu^1,2^ , Po-Jung Tseng ^4^_,_ Kuo-Liong Chien*^3,5^**

^1^ Division of Endocrinology and Metabolism, Department of Internal Medicine, Mackay Memorial Hospital, Taipei, Taiwan

^2^ Department of Medicine, Mackay Medical Collage, New Taipei City, Taiwan

^3^ Institute of Epidemiology and Preventive Medicine, College of Public Health, National Taiwan University, Taipei, Taiwan

^4^ Division of Cardiovascular Surgery, Department of Surgery, Hsin Chu Armed Force Hospital, Hsinchu, Taiwan

^5^ Department of Internal Medicine, National Taiwan University Hospital, Taipei, Taiwan

*Corresponding authors:

Kuo-Liong Chien, MD., PhD.

Institute of Epidemiology and Preventive Medicine, College of Public Health, National Taiwan University, Taipei, Taiwan

E-mail: klchien@ntu.edu.tw

Supplemental Table Legend

Supplemental Table 1. The search terms for the search strategy in PubMed, EMBASE, Cochrane Library, and EBSCO

Supplemental Table 2. Inclusion and exclusion criteria

Supplemental Table 3 Characteristics of participants in 20 cohort studies

Supplemental Table 4 Bias Assessment: Risk of Bias in Non-randomized Studies of Interventions (ROBINS-I)

Supplemental Table 5 Baseline characteristics as effect modifier factors between the association of combined healthy lifestyle factors and CVD reduction from the univariate and multivariate meta-regression models

Supplemental Table 6. Multivariate meta-regression model considering age and women proportion with and without a cross-product term

Supplemental Figure Legend

Supplemental Figure 1. The process of literature search based on the PRISMA statement

Supplemental Figure 2 Funnel Plot

Supplemental Figure 3 Contour-enhanced Funnel Plot

Supplemental Figure 4 Trim and Fill

Supplemental Figure 5 Egger Test:

Supplemental Figure 6. Forest plot of adjusted hazard ratios with corresponding 95% CIs of those with the maximal numbers of healthy lifestyle compared to those with the minimal numbers of healthy lifestyle and the incidence of coronary heart disease (A), ischemic stroke (B), heart failure (C), cardiovascular disease (D) as outcome measurement.

Supplemental Figure 7. Subgroup analysis for the age subgroup 37.1-49.9 years, 50.0–59.9 years, and 60.0–72.9 years of adjusted hazard ratios with corresponding 95% CIs of those with the maximal numbers of healthy lifestyle compared with those with the minimal numbers of healthy lifestyle and the incidence of cardiovascular disease

Supplemental Figure 8. Forest plot of adjusted hazard ratios with corresponding 95% CIs of those with the maximal numbers of healthy lifestyle compared to those with the minimal numbers of healthy lifestyle and the incidence of coronary heart disease stratified by ethnic groups

Supplemental Figure 9 The bubble plot of baseline characteristics as effect modifier factors between the association of combined healthy lifestyle factors and CVD reduction from the univariate meta-regression model

Supplemental Figure 10. The hazard ratio from combined healthy lifestyle factors on cardiovascular diseases by a cumulative meta-analysis

**Supplemental Material**

Supplemental Table 1. The search terms for the search strategy in PubMed, EMBASE, Cochrane Library, and EBSCO

| **Lifestyle** | **Score** | **CV risk and cardiac death** | **Study** |
| --- | --- | --- | --- |
| Lifestyle | Combined | Cardiac death | Longitudinal |
| Lifestyles | Combined effects | Death, sudden, cardiac | Cohort |
| Lifestyle | Combination | Cardiovascular disease | Prospective |
| Lifestyles | Joint impact | Cardiovascular disorder |  |
| Healthy lifestyle | Joint effects | Intracranial vascular disease |  |
| Healthy lifestyle | Joint association | Vascular diseases, intracranial |  |
| Risky health behaviors | Integrated | Intracranial vascular disorder |  |
| Risky health behaviors | Adherence | Brain vascular disorder |  |
| Risky health behaviors | Adhering | Stroke |  |
| Modifiable factors | Pattern | Cerebrovascular accident |  |
| Recommendations | Score | Cerebrovascular apoplexy |  |
| Protective factors | Index | CVA |  |
| Primary prevention | Indices | Myocardial ischemia |  |
| Health promotion |  | Ischemic heart disease |  |
|  |  | Coronary artery disease |  |
|  |  | Heart failure |  |
|  |  | Cardiac failure |  |
|  |  | Heart decompensation |  |
|  |  | Myocardial failure |  |
|  |  | Myocardial infarction |  |
|  |  | Heart attack |  |
|  |  | Myocardial infarcts |  |
|  |  | Cerebral stroke |  |

Supplemental Table 2. Inclusion and exclusion criteria

|  | Inclusion criteria | Exclusion criteria |
| --- | --- | --- |
| Population | Adults overo 18 years of age | With history of coronary heart disease, stroke, heart failure or myocardial infarction |
| Exposure | A combination of at least 3 lifestyle factors | A single or a combination of only two lifestyle factors |
| Outcome | The hazard ratio of cardiovascular disease including coronary heart disease (CHD), stroke, heart failure (HF), or myocardial infarction (MI) | The hazard ratios of cardiovascular disease are not reported separately or the effective measurements are not provided separately |
| Study design | Cohort Studies | Randomized controlled trials, randomized clinical trials or clinical trials, cross-sectional, case-control, systematic reviews and meta-analysis, protocols, clinical case, editor letters, qualitative studies and observational studies |
| Language | English | Other language |

Supplemental Table 3: Characteristic of participants in 20 cohort studies

| Reference | Cohort name (Country), follow-up years | Study population: participants (women proportion); age at baseline (mean age); DM prevalence | Components of the lifestyle score | Events, outcome measurement, | Adjustment factors |
| --- | --- | --- | --- | --- | --- |
| Stampfer,  2000 | Nurses’ Health Study (U.S.), mean follow-up: 14 years | 84129 women; age: 30~55 years; 0% DM | Smoking habit, physical activity, alcohol intake, diet (trans fat, glycemic load, fiber, unsaturated to saturated fat) | 1128, Fatal myocardial infarction, non-fatal myocardial infarction | Age, time period (seven time periods), parental history of myocardial infarction before the age of 60 years, menopausal status, postmenopausal hormones, hypertension, TC |
| Akesson,  2007 | Swedish Mammography Cohort (Sweden), mean follow-up: 6.2 years | 24444 women,  age: 48-83 years (mean:59.2); 0% DM | Diet (healthy dietary pattern score), alcohol | 308, Myocardial infarction, Death | Age, educational, family history of myocardial infarction, TC, hypertension, hormone therapy, aspirin, total energy intake |
| Myint,  2009 | Norfolk cohort of European Prospective Investigation of Cancer (EPIC Norfolk), mean follow-up: 11.5 years | 20040 men and women (55.3%); age: 40-79 years, (mean:58.3); 1.9% DM | Smoking habit, physical activity, alcohol intake, diet (fruit and vegetable intake) | 599, Stroke | Age, sex, BMI, systolic blood pressure, TC, diabetes, aspirin, and social class |
| Ford,  2009 | European Prospective Investigation into Cancer and Nutrition (EPIC)-Potsdam study (German), mean follow-up: 7.8 years | 23,153 women;  age: 35-65 years,  men: 40 - 65 years; women: 35 - 65 years (mean:49.3); 0% DM | Smoking, BMI, physical activity, diet (consumption of fruits and vegetables, whole grain bread, red meat) | 214, Myocardial infarction | Age, sex, education, family history of stroke, diabetes, systolic BP, TC |
| Djousse,  2009 | Physicians' Health Study I (U.S.), mean follow-up: 22.4 years | 20,900 men; (mean age:53.6); 2% DM | BMI, smoking, exercise, alcohol consumption, diet (breakfast cereals, vegetables, fruits) | 1200, Heart failure | No specific mention |
| Zhang,  2011 | FINRISK (Finland)  , mean follow-up: 13.7 years | 36,686 men and women (53% women); age: 25-74years (mean: 45.8); 2.2% DM | Smoking, BMI, physical activity, diet (vegetable consumption), alcohol consumption | 1167, Ischemic stroke | age, sex, education, family history of stroke, diabetes, systolic BP, TC |
| Wang,  2011 | FINRISK (Finland)  , mean follow-up: 14.1 years | 38,075 men and women (51.8%); age: 25-42 years (mean: 46.7); 2.5% DM | Smoking, BMI, physical activity, diet (vegetable consumption) | 1083, Heart failure | Age, study year, education, systolic BP, TC, histories of myocardial infarction, valvular heart disease, diabetes, hypertension medication, |
| Carlsson,  2013 | Population-based prospective cohort study in Stockholm (Sweden), mean follow-up: 10.85 years | 4232 men and women (51.8%); (mean age: 60 years old); 6.8% DM | Smoking, alcohol intake, leisure time physical activity, diet (consumption of fish, processed meats, fruits, vegetables) | 375, Ischemic cardiovascular diseases | Sex, educational level, BMI |
| Hoevenaar-Blom, 2014 | Monitoring Project on Risk Factors for Chronic Diseases (MORGEN) (Netherlands), mean follow-up: 12 years | 14,639 men and women (54.42 %); age: 20-65 years (mean:41.5); 0% DM | Physical activity, diet (MDS), alcohol consumption, smoking, sleep duration | 607, Fatal and non-fatal cardiovascular diseases | Age, sex, educational level, BMI, systolic blood pressure, and TC/HDL ratio |
| Ahmed,  2013 | Multi-Ethnic Study of Atherosclerosis (MESA)(U.S.), mean follow-up: 10 years | 6229 men and women (53%); age: 44-84 years (mean:10); 10% DM | Diet (Mediterranean-style versus unhealthy diet), BMI, smoking status, physical activity | 655, Coronary cardiovascular events | Hypertension, hypertension medication, diabetes medication, lipid-lowering medications, fasting plasma glucose, HDL, non–HDL, TG, CRP |
| Paynter,  2014 | Women's Health Initiative Observational Study (WHI-OS) (U.S.), mean follow-up: 10 years | 60890 women, Age: 50-79 years (mean:10); 3.5% DM | BMI or WC, physical activity, alcohol smoking (all participants by inclusion criteria), Diet (AHEI), | 1808, Major cardiovascular disease | Age, smoking status, family history of a premature MI, hemoglobin A (if participants had a history of diabetes mellitus), high sensitivity CRP, systolic blood pressure, total and HDL cholesterol, race |
| Larsson,  2014 | Swedish Mammography Cohort (Sweden), mean follow-up: 10.4 years | 31696 women; age: 49-83 years (mean:10.4); 3.38% DM | alcohol smoking, physical activity, BMI Diet (RFS), | 1155, Ischemic stroke | age, education, aspirin use, diabetes, family with atrial fibrillation and myocardial infarction before 60 years of age, total energy intake, and Non- Recommended Food Score. |
| Akesson,  2014 | Cohort of Swedish Men (Sweden), mean follow-up: 11 years | 20721 men; age: 45-79 years old, (mean: 58.6); 0% DM | Alcohol smoking, physical activity, abdominal adiposity (WC) diet (RFS), | 1361, Myocardial infarction | Age, educational level, family with myocardial infarction, aspirin, marital status, non-Recommended Food Score, and total energy intake |
| Agha,  2014 | Women's Health Initiative Observational Study (WHI-OS) (U.S.), mean follow-up: 11 years | 84,537 Women; (mean age :11); 3.86% DM | physical activity, smoking, BMI diet (AHEI), | 1826, Heart failure | Age, race/ethnicity, marital status, education, U.S. region, antecedent CHD, treated diabetes, hypertension |
| Del Gobbo, 2015 | Cardiovascular Health Study (U.S.), mean follow-up: 21.5 years | 4,490 men and women ( 61%); age:≧65 years; (mean:21.5) | Physical activity (walking pace + leisure activity), alcohol use, smoking, adiposity (BMI) | 1380, Heart failure | Age, sex, race, BMI, hypertension medication, diabetes, and baseline CHD, |
| Chomistek, 2015 | Nurses' Health Study II cohort (U.S.), mean follow-up: 20 years | 88940 women; age: 27-44 years (mean:20); 0% DM | Smoking, physical activity, TV watching, BMI, alcohol, diet (AHEI-2010), | 456, Coronary heart disease | Age, time period, parental history of MI before 60 years of age, aspirin, menopausal status, postmenopausal hormone, parity, and oral contraceptive |
| Larsson,  2016 | Cohort of Swedish Men and Swedish Mammography Cohort (Sweden), mean follow-up: 13 years | 33,966 men and women (53%); age: 45-83 years (mean:13); 7.4% DM | Smoking, physical activity, BMI, diet (modified Mediterranean diet) | 1488, Heart Failure |  |
| Lv,  2017 | Kadoori Biobank cohort (China), mean follow-up: 7.2 years | 461,211 men and women (59%); age: 30-79 years (mean:50.7); 0% DM | Smoking, alcohol, physical activity, diet (fruits, vegetables, meat consumption), BMI, WHR | 3331, Major coronary events death and nonfatal myocardial infarction and ischemic stroke | age, sex, education, marital status, and family histories of heart attack or stroke |
| Díaz-Gutiérrez, 2017 | Seguimiento Universidad de Navarra (SUN) cohort (Spain), mean follow-up: 10.4 years | 19,336 men and women ( 61.4%); age: ≧18 years (mean:37.3); 1.7% DM | Smoking, physical activity, BMI, alcohol, diet (Mediterranean diet), TV watching, binge drinking, having short afternoon nap, time with friends, time working | 140, cardiovascular disease | Age, sex, year questionnaire completion, diabetes, cardiovascular disease (other than acute coronary syndrome and stroke), hypertension, hypercholesterolemia, hypertriglyceridemia |
| Dimovski,  2019 | Malmö Diet and Cancer study (MDCS) (Sweden), mean follow-up: 18 years | 26,323 men and women (61.9%); age: 43-73 years (mean: 57.6); 4.0% DM | Smoking, BMI, physical activity, healthy diet | 3417, Coronary artery disease, death due to ischemic heart disease, percutaneous coronary intervention, or coronary artery bypass grafting | Age, sex, educational level and parental history of MI |

Supplemental Table 4

Bias Assessment: Risk of Bias in Non-randomized Studies- of Interventions (ROBINS-I)

|  | confounding | selection of participants into the study | classification of exposures | deviations from intended exposures | missing data | measurement of outcomes | selection of the reported result | Risk of bias scores |
| --- | --- | --- | --- | --- | --- | --- | --- | --- |
| Myint, 2009 | Moderate | Low | Low | NI | Moderate | Low | Low | 3 |
| Stampfer,2000 | Low | Low | Low | Low | Low | Low | Low | 0 |
| Akesson, 2007 | Low | Low | Low | NI | Low | Low | Low | 1 |
| Ford, 2009 | Moderate | Low | Low | Low | Low | Moderate | Low | 2 |
| Djousse, 2009 | Moderate | Moderate | Low | Low | Low | Moderate | Low | 3 |
| Cardi, 2009 | NI | Moderate | Low | NI | NI | Low | Low | 4 |
| Zhang, 2011 | Low | Low | Low | Low | Low | Low | Low | 0 |
| Wang, 2011 | Low | Low | Low | Low | Low | Low | Low | 0 |
| Hoevenaar-Blom, 2013 | Moderate | Low | Low | NI | Low | Low | Low | 2 |
| Carlsson, 2013 | Moderate | Low | Moderate | NI | Low | Low | Low | 3 |
| Ahmed, 2013 | Low | Moderate | Low | NI | Moderate | Moderate | Low | 4 |
| Paynter, 2014 | Low | Moderate | Low | NI | Moderate | Moderate | Low | 4 |
| Larsson, 2014 | Low | Moderate | Low | Low | Low | Low | Low | 1 |
| Akesson, 2014 | Low | Low | Low | NI | Moderate | Low | Low | 2 |
| Agha, 2014 | Moderate | Low | Low | NI | Low | Low | Low | 2 |
| Del Gobbo, 2015 | Moderate | Low | Low | Low | Low | Low | Low | 1 |
| Chomistek, 2015 | Low | Low | Low | Low | Low | Moderate | Low | 1 |
| Larsson, 2016 | Low | Low | Low | NI | Moderate | Low | Low | 2 |
| Lv, 2017 | Low | Low | Low | NI | Low | Low | Low | 1 |
| Díaz-Gutiérrez, 2017 | Moderate | Low | Low | NI | Low | Moderate | Low | 3 |
| Dimovski,2019 | Low | Low | Low | NI | Low | Low | Low | 1 |

Supplemental table 5

Baseline characteristics as effect modifier factors between the association of combined healthy lifestyle factors and CVD reduction from the univariate and multivariate meta-regression model. (A. according to the prevalence of diabetes mellitus in the study participants at baseline; B. according to the follow-up duration in the original studies; C. according to the proportion of women); D. according to the ethnic groups (American as reference)

|  | Slope | 95% CI | | τ^2^ (%) | I^2^ (%) | p value |
| --- | --- | --- | --- | --- | --- | --- |
| Univariate model | >-0.01 | -0.08 | 0.06 | 2.44 | 27.52 | 0.79 |
| Multivariate model* | -0.02 | -0.06 | 0.01 | 0.38 | 5.68 | 0.23 |

B.

|  | Slope | 95% CI | | τ^2^ (%) | I^2^ (%) | p value |
| --- | --- | --- | --- | --- | --- | --- |
| Univariate model | >-0.01 | -0.05 | 0.05 | 4.06 | 41.32 | 0.93 |
| Multivariate model* | -0.02 | -0.06 | 0.01 | 0.38 | 5.68 | 0.23 |

C.

|  | Slope | 95% CI | | τ2 (%) | I2 (%) | p value |
| --- | --- | --- | --- | --- | --- | --- |
| Univariate | 0 | -0.004 | 0.004 | 2.85 | 34.24 | 0.986 |
| Multivariate* | >-0.001 | -0.005 | 0.004 | 1.61 | 22.14 | 0.79 |

D.

|  | Slope | 95% CI | | p value |
| --- | --- | --- | --- | --- |
| Asia | -0.43 | -1.58 | 0.72 | 0.44 |
| European | -0.34 | -0.73 | 0.06 | 0.09 |

Note:

Multivariate model adjusted with age and sex;

τ^2^: the variance of the true effect sizes QM statistic and its p value show whether the moderator is statistically significant in explaining heterogeneity

Supplemental Table 6. Multivariate meta-regression model considering age and women proportion with and without a cross-product term (A. Without a cross-product term of age and women proportions; B. With a cross-product term of age and women proportions)

|  | Slope | 95% CI | | p value |
| --- | --- | --- | --- | --- |
| Intercept | -1.79 | -2.59 | -0.99 | <0.001 |
| Age (years old) | 0.01 | <0.001 | 0.03 | 0.042 |
| Women (%) | >-0.001 | -0.005 | 0.004 | 0.79 |

B.

|  | Slope | 95% CI | | p value |
| --- | --- | --- | --- | --- |
| Intercept | -0.4 | -2.78 | 1.97 | 0.72 |
| Age (years old) | -0.01 | -0.05 | 0.03 | 0.63 |
| Women (%) | -0.02 | -0.06 | 0.01 | 0.21 |
| Age*Women | <0.001 | >-0.001 | 0.001 | 0.22 |

Note. Multivariate models: A. age and women proportion; B. age, women proportion and cross-product term of age and women proportion.

Supplemental Figure 1. The process of literature search based on the PRISMA statement

Records identified initially by PubMed (n = 14862), EMBASE (n = 4515), Cochrane Library (n = 809) and EBSCO (n = 662), total n = 20248

Identification

Studies excluded due to duplicated (n = 1007)

Screening

Examined studies by title (n = 19241)

Studies excluded based on title (n = 17815)

Examined studies by abstract (n = 1426)

Excluded non-relevant abstracts (n = 1325)

- Topic not of interest (n = 770)
- The population with chronic disease (n = 251)
- Intervention study (n = 148) or review (n = 156)

Full-text articles were evaluated for eligibility (n = 101)

Eligibility

Excluded based on full-text (n = 74)

- Combined other risk factors than lifestyle (n = 10)
- Combined less than 3 lifestyles factors (n = 9)
- Definition of interest not compatible with ours (n = 1)
- No CVD endpoint (n = 13), only mortality (n = 40)
- Ascertainment of cardiovascular diseases not compatible with ours(n = 1)

Included

Studies included in systemic review (n = 27)

Excluded after systemic review (n = 7)

- Studies excluded due to lack of HR and 95% CI (n = 2)
- Duplicated publication of the same cohort not in overall meta-analysis but in subgroups (n = 5)

Studies included in quantitative synthesis (overall meta-analysis) (n = 20)

Supplemental Figure 2: Funnel Plot


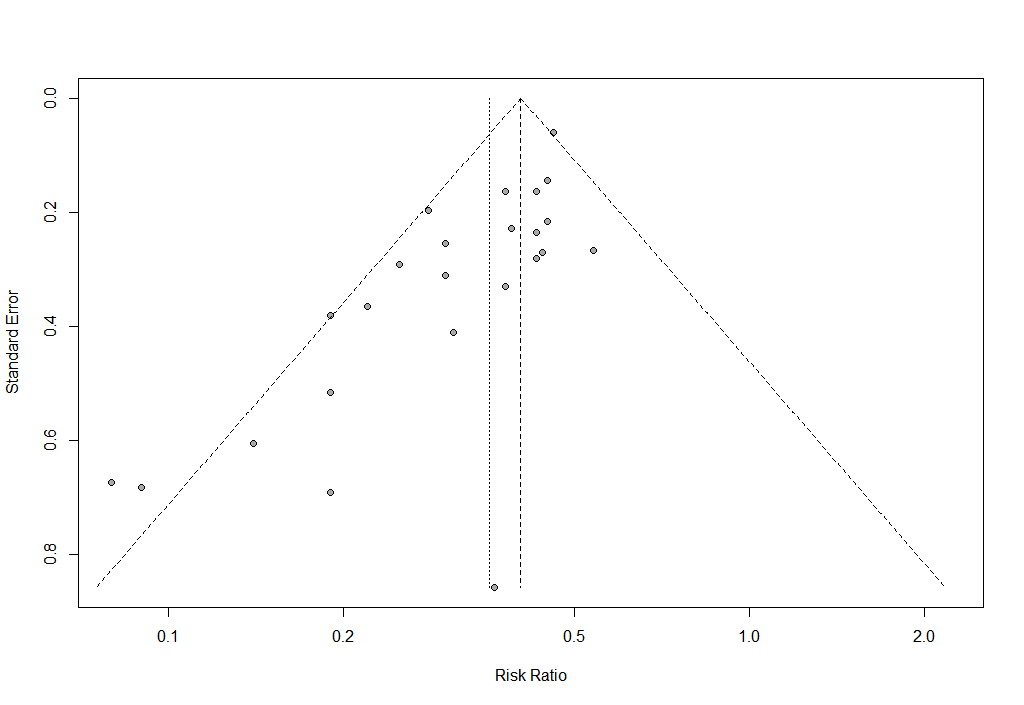


Supplemental Figure 3: Contour-enhanced Funnel Plot


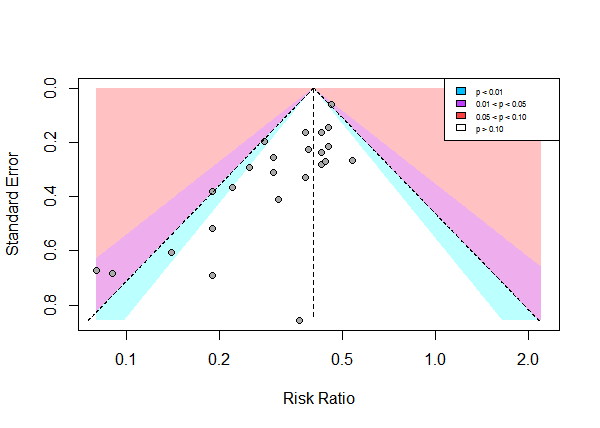


Supplemental Figure 4: Trim and Fill


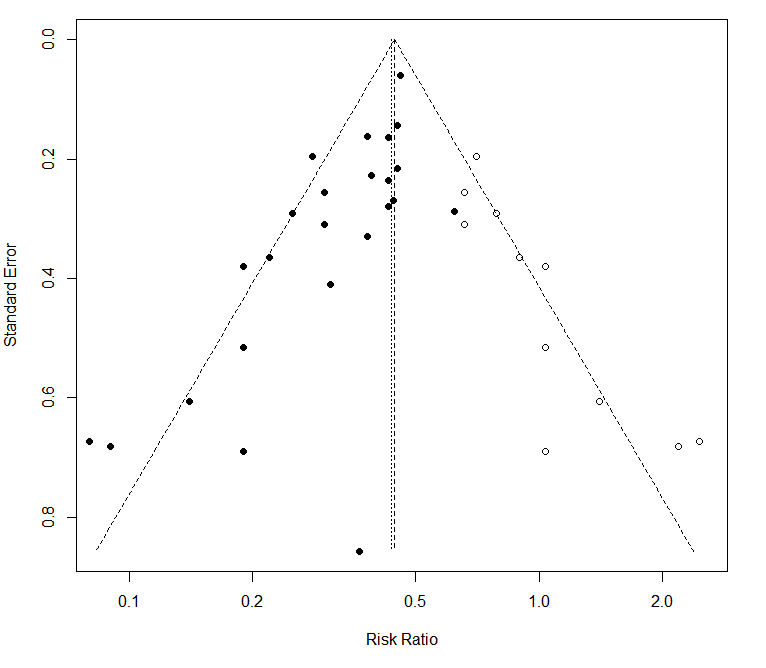


Supplemental Figure 5: Egger Test:


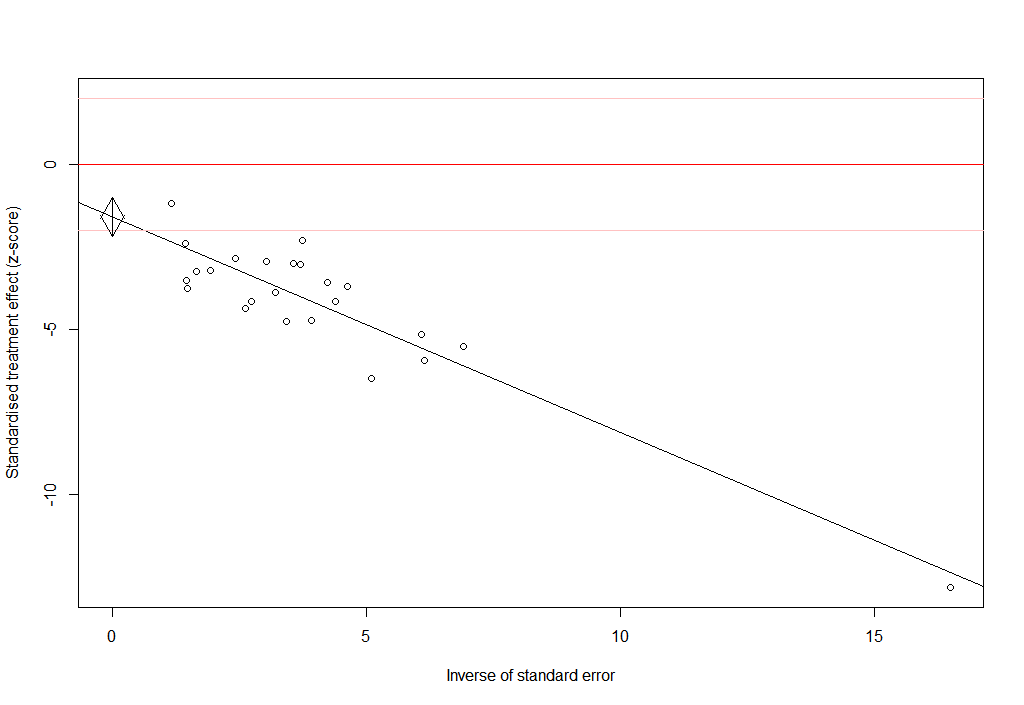


Supplemental Figure 6. Forest plot of adjusted hazard ratios with corresponding 95% CIs of those with the maximal numbers of healthy lifestyle compared to those with the minimal and the incidence of coronary heart disease (A), ischemic stroke (B), heart failure (C), cardiovascular disease (D) as outcome measurement.

Figure 6.A


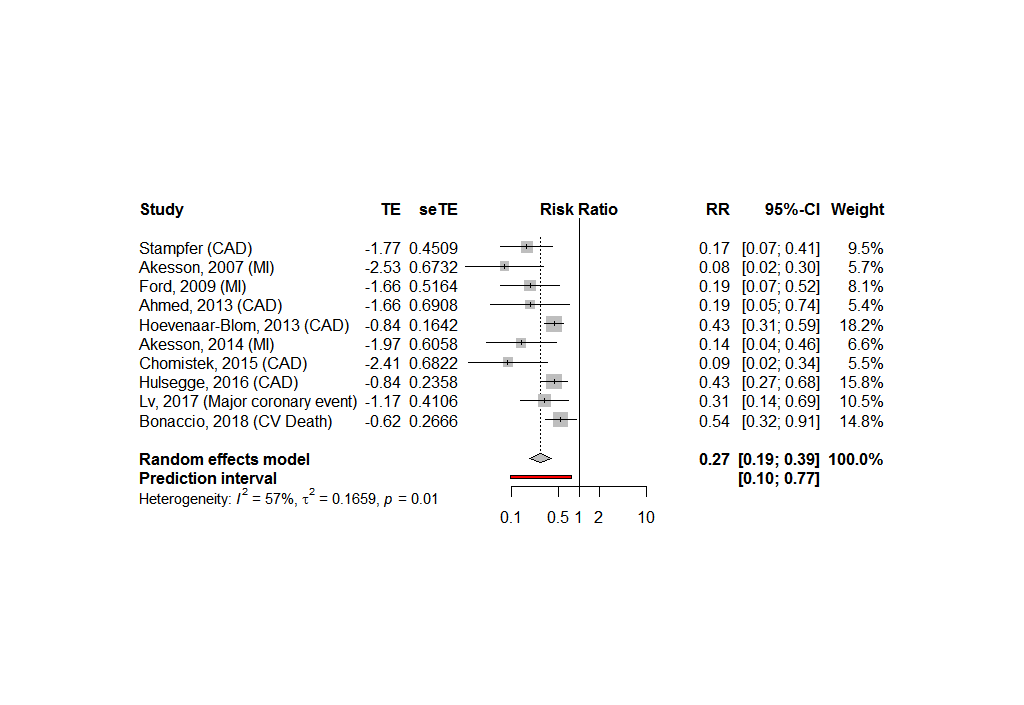


Figure 6.B


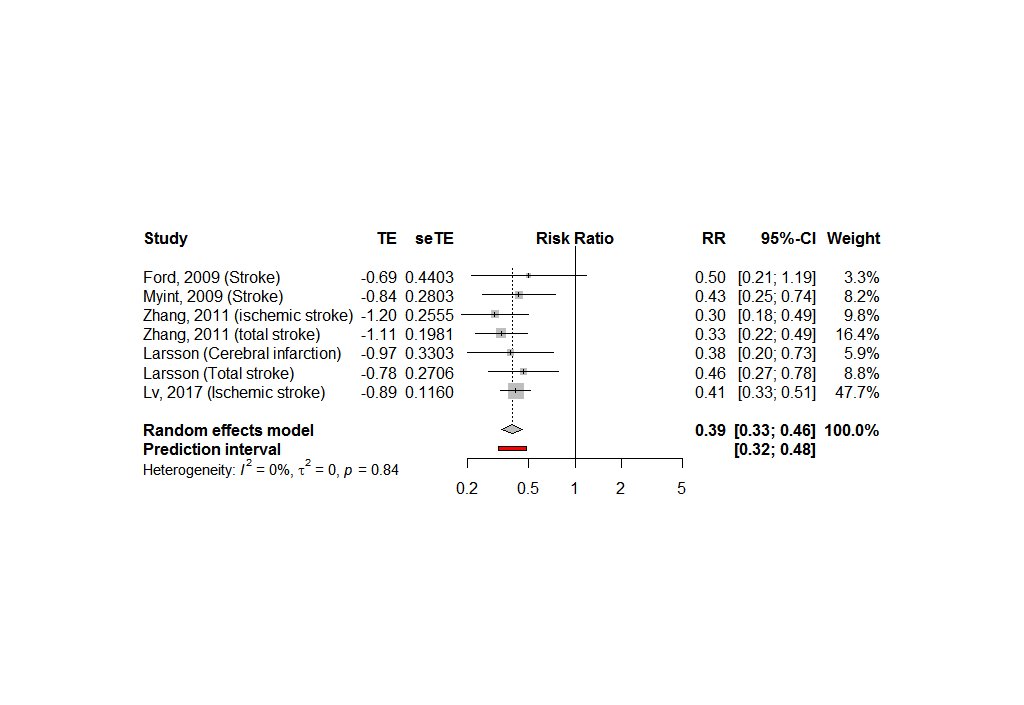


Figure 6.C


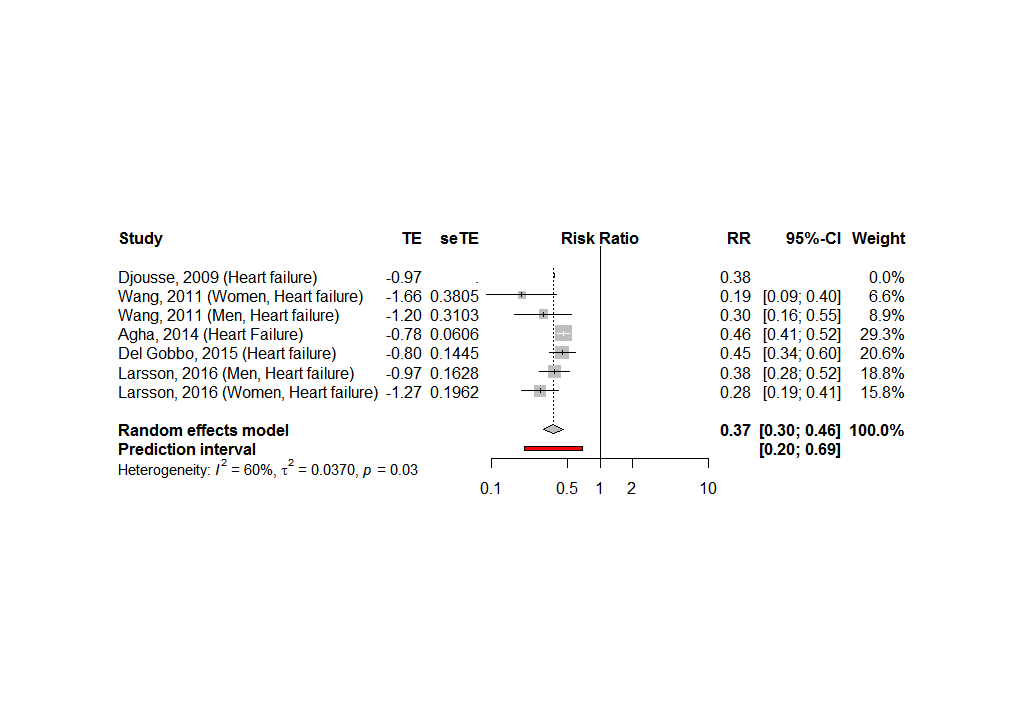


Figure 6.D


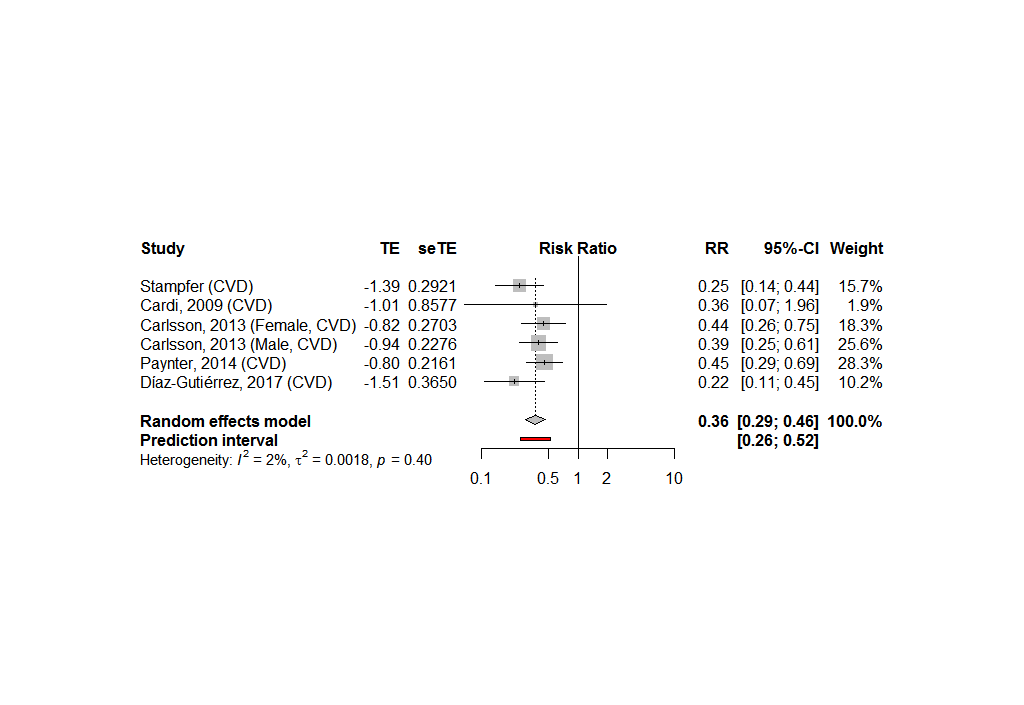


Supplemental Figure 7. Subgroup analysis for the age subgroup 37.1-49.9 years; 50.0–59.9 years; 60.0–72.9 years of adjusted hazard ratios with corresponding 95% CIs of those with the maximal numbers of healthy lifestyle compared with those with the minimal and the incidence of cardiovascular disease (A) coronary artery disease stratified; (B) stroke; (C) heart failure

A.


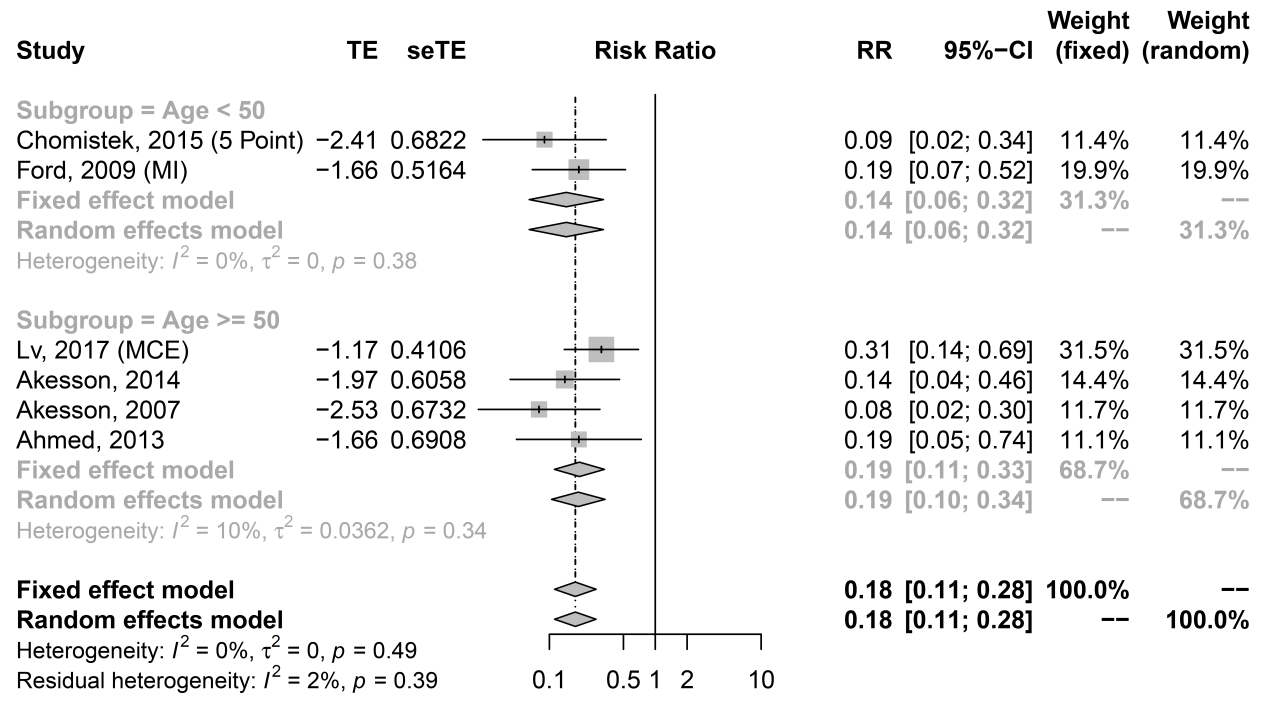


B.


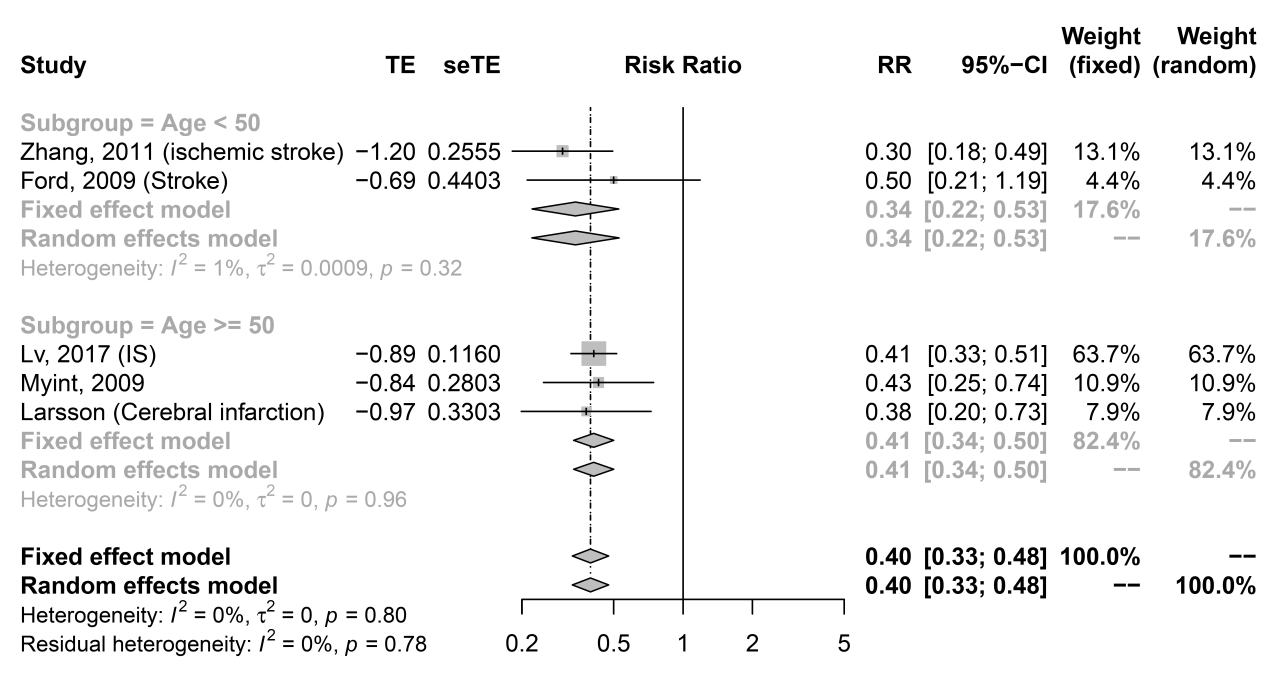


C.


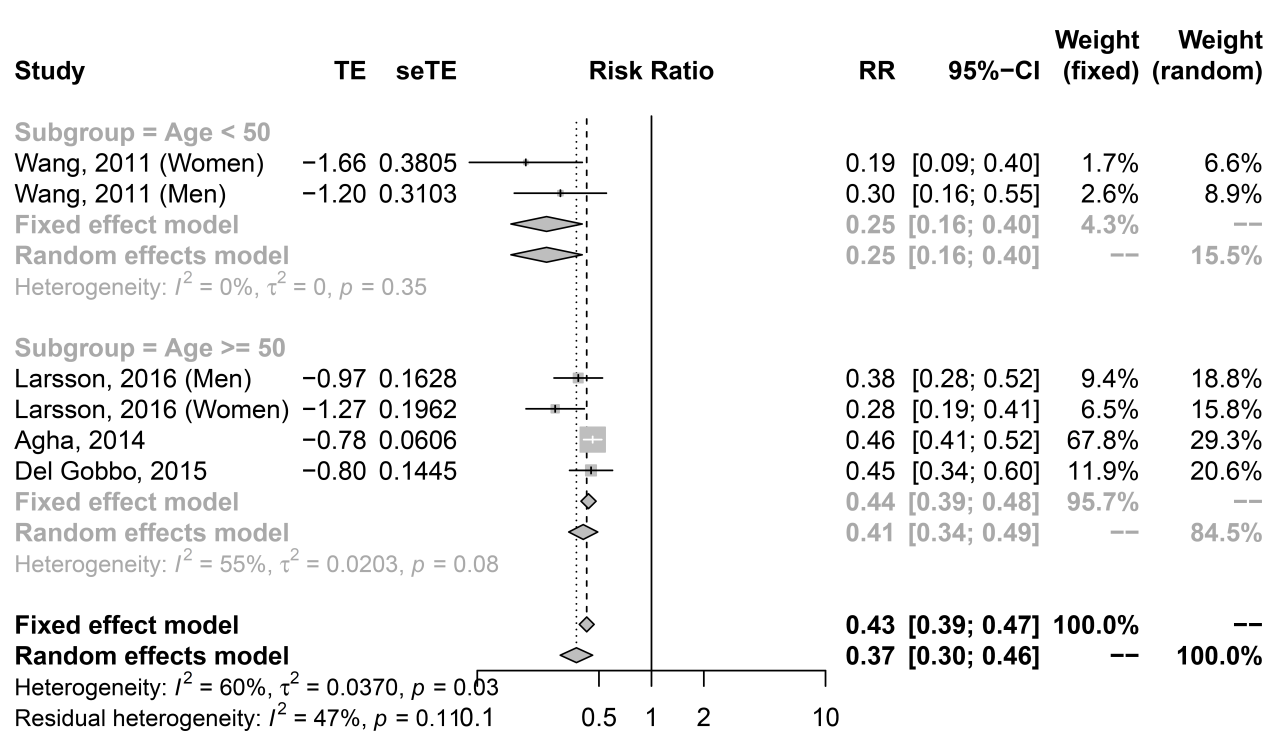


Supplemental Figure 8. Forest plot of adjusted hazard ratios with corresponding 95% CIs of those with the maximal numbers of healthy lifestyle compared to those with the minimal and the incidence of coronary heart disease stratified by ethnic groups (A), American (B), European (C), Asia.


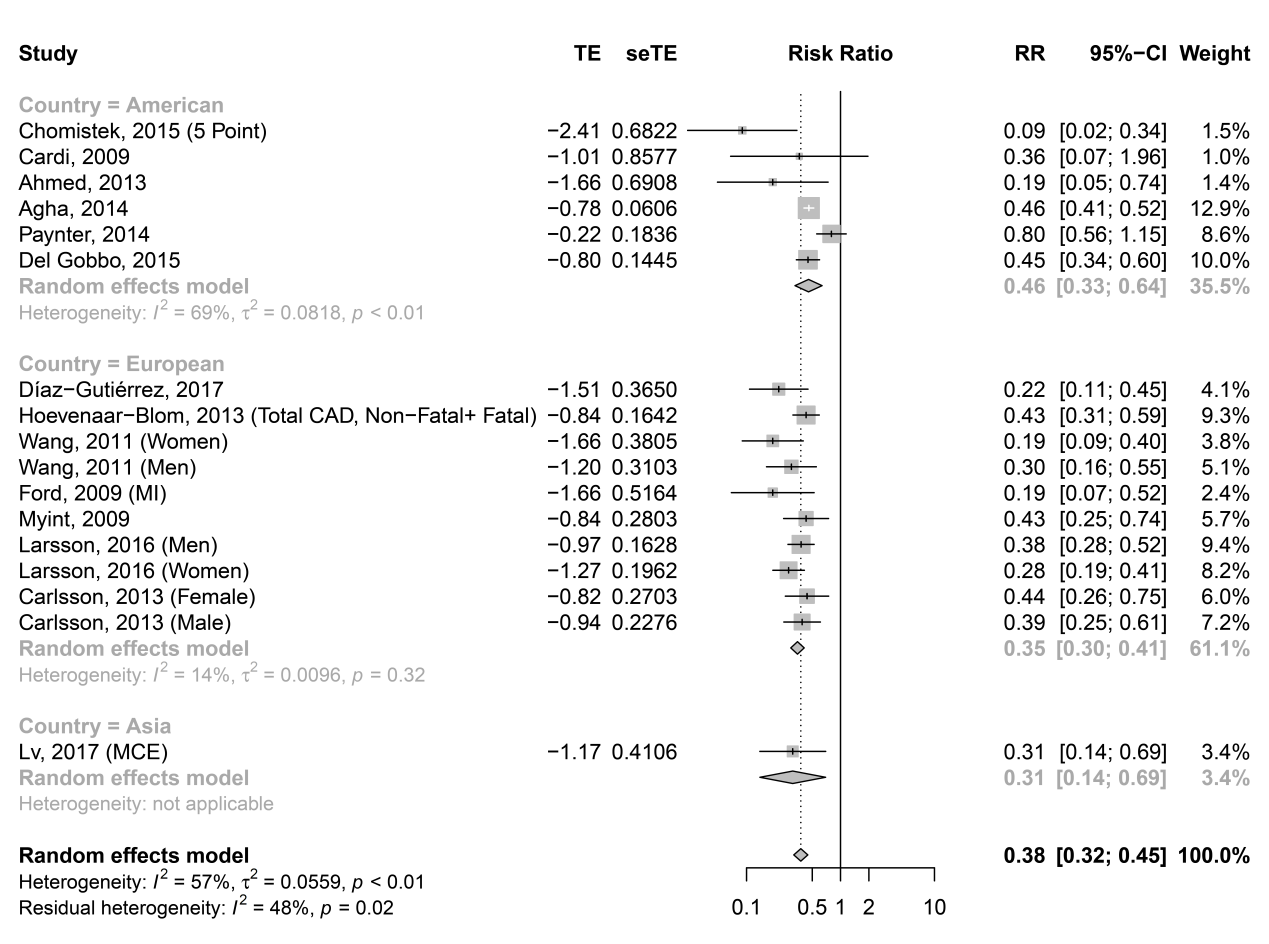


Supplemental Figure 9 The bubble plot of baseline characteristics as effect modifier factors between the association of combined healthy lifestyle factors and CVD reduction from the univariate meta-regression model. (A. according to the prevalence of diabetes mellitus in the study participants at baseline; B. according to the follow-up duration in the original studies; C. according to the proportion of women)

(A)

The prevlanece of Diabetes Mellitus


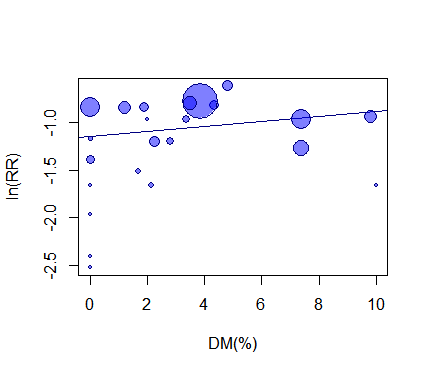


Risk of cardiovascular disease

(%)

(B)

Risk of cardiovascular disease

The follow-up uration


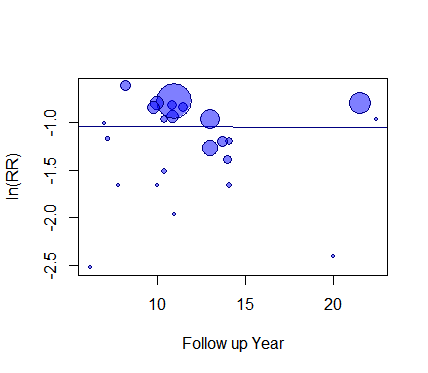


(Years)

(C)

The proportion of women


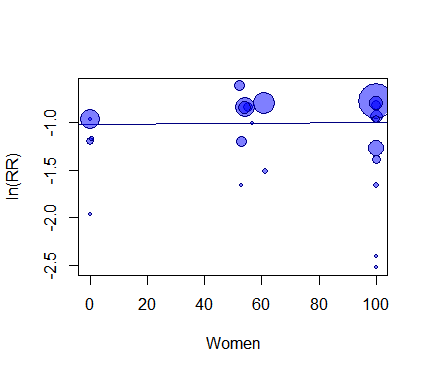


Risk of cardiovascular disease

(%)

Supplemental Figure 10. The hazard ratio from combined healthy lifestyle factors on cardiovascular diseases by a cumulative meta-analysis.


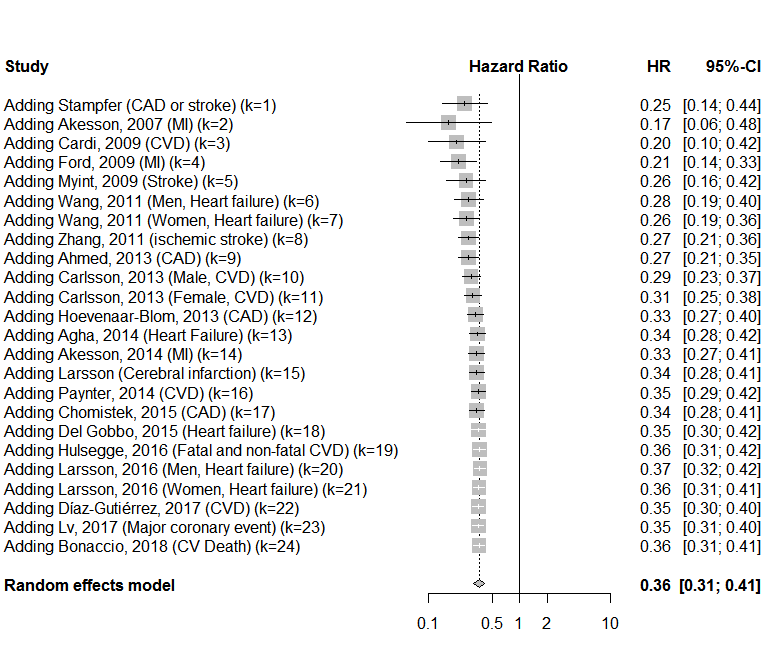

Supplement: Supplementary file 1 — Supplementary Informations. [file 41598_2020_75314_MOESM1_ESM.docx]
